# A Randomized Multicenter Study Comparing a Tacrolimus-Based Protocol with and without Steroids in HCV-Positive Liver Allograft Recipients

**DOI:** 10.1155/2012/894215

**Published:** 2012-05-28

**Authors:** Ulf Neumann, Didier Samuel, Pavel Trunečka, Jean Gugenheim, Giorgio Enrico Gerunda, Styrbjörn Friman

**Affiliations:** ^1^Department of General, Visceral and Transplantation Surgery, Charité, Campus Virchow Clinic, 13353 Berlin, Germany; ^2^Chirurgische Klinik, Universitätsklinikum Aachen, 52074 Aachen, Germany; ^3^Centre Hépato-Billaire, Hôpital Paul Brousse, 94804 Villejuif, France; ^4^Department of Hepatogastroenterology, IKEM, 140 21 Praha, Czech Republic; ^5^Service de Chirurgie Générale et Digestive, Hopital l'Archetet II, 6000 Nice, France; ^6^Centro Trapianti Multiviscerale, di Fegato e di Chirurgia Epatobiliopancreatica, Azienda Ospedaliero Universitaria di Modena, 40124 Modena, Italy; ^7^Transplant Institute, Sahlgrenska University Hospital, 413 45 Göteborg, Sweden

## Abstract

Allograft reinfection with hepatitis C virus (HCV) occurs universally in liver transplant recipients. Corticosteroids can contribute to HCV recurrence. This randomized study evaluated HCV recurrence in HCV-positive liver allograft recipients using steroid-free immunosuppression. All patients received tacrolimus (TAC) at an initial dose of 0.10–0.15 mg/kg. The steroid-free arm (TAC/daclizumab (TAC/DAC, *n* = 67)) received daclizumab induction, and the steroid arm (TAC/steroid (TAC/STR, *n* = 68)) received a steroid bolus (≤ 500mg) followed by 15–20 mg/day with discontinuation after month 3. Median HCV viral load at month 12, the primary endpoint, was similar at 5.46 (0.95–6.54) IU/mL with TAC/DAC and 5.91 (0.95–6.89) IU/mL with TAC/STR. Small numerical differences in the estimated rate of freedom from HCV recurrence (19.1 versus 13.8%) and freedom from biopsy proven rejection (78.4 versus 66.1%) were observed between TAC/DAC and TAC/STR. Patient survival estimates were significantly lower with TAC/DAC than with TAC/STR (83.1 versus 95.5%; 95% CI, −0.227 to −0.019%), and graft survival was numerically lower (80.1 versus 91.1%, *P* = NS). Completion rates (45 versus 82%) indicated poorer tolerability with TAC/DAC than with TAC/STR. Steroid-free immunosuppression had no real impact on HCV viral load. HCV recurrence was higher with TAC/STR. Results are inconclusive due to the unexpected lower completion rates in the TAC/DAC arm.

## 1. Introduction

Cirrhosis secondary to hepatitis C virus (HCV) is the most common indication for orthotopic liver transplantation. Unfortunately, allograft reinfection with HCV occurs universally in liver transplant recipients. Acute recurrence can occur within 6 months after transplantation [1], is often more severe than the primary HCV disease, and leads to fairly rapid progression to cirrhosis. Recurrent viral infection with progression to cirrhosis and graft failure is the most frequent cause of morbidity in the posttransplant setting [2–4].

Factors possibly contributing to the recurrence of HCV include viral HCV-related factors (viral load and genotype [5, 6]), coinfection with other viruses, donor-related factors, type and amount of immunosuppression, and steroid pulses for treatment of acute rejection [7, 8].

The choice of calcineurin inhibitor does not seem to influence HCV recurrence [9]. In a prospective randomized study, no significant difference in HCV recurrence or HCV progression was found between tacrolimus- and cyclosporine-based treatments [5]. A relationship between steroids and the severity of HCV recurrence has, however, been observed [10]. Posttransplant tapering of steroids has been found to reduce the progression of recurrent HCV [11, 12] while pulse administration of steroids for treatment of acute rejection has been associated with development of cirrhosis [13]—a primary cause of graft loss in liver transplantation.

In this study we explored the impact of steroid-free immunosuppression on HCV viral load at 12 months in patients transplanted for HCV cirrhosis. The onset of HCV-related liver disease is difficult to determine as the clinical signs and symptoms of HCV are similar to those of acute rejection and the two conditions can coexist [14]. We therefore used HCV viral load as a surrogate indicator of HCV recurrence because of the potential difficulty in differentiating between acute rejection and HCV disease after transplantation. To test the effect of a steroid-free regimen, we compared two tacrolimus-based protocols: one with steroid administration for 3 months (the reference treatment) and the other with daclizumab in which steroids were avoided for both prophylactic immunosuppression, and wherever possible, antirejection treatment (experimental treatment): we assumed that HCV recurrence would be lower with the steroid avoidance regimen. Safety and efficacy evidence for omitting steroids [15, 16] and for replacing steroids with daclizumab [17] in immunosuppression protocols in liver transplantation has been demonstrated in randomized multicenter clinical trials.

## 2. Methods

### 2.1. Study Design and Patients

This was a prospective, randomized, open-label, parallel arm study that was conducted between June 2005 and June 2008 at 17 centers in 8 European countries. Patients were followed up to 12 months unless they withdrew consent or withdrew from treatment for reasons other than death or graft loss. Inclusion criteria included age above 18 years, hepatitis C virus positive, and first orthotopic (whole or split) liver transplant. Exclusion criteria were on-going steroid administration, HIV positivity, ABO incompatibility, and a previous history of malignancy other than treated nonmelanoma skin cancer. Patients with hepatocellular carcinoma were included unless they had >3 nodules, the nodules were >5 cm in diameter, and there was evidence of vascular invasion, metastases, or local invasion.

The study was conducted in compliance with the Declaration of Helsinki and Good Clinical Practice guidelines and in accordance with local and national regulatory requirements and laws. All relevant study documents were approved by the Institutional Review Board responsible for the study center. All patients provided signed informed consent and could withdraw from the study at any time.

### 2.2. Treatment Intervention

Tacrolimus was administered to patients in both treatment arms. The initial daily dose was 0.10–0.15 mg/kg. Recommended trough levels from day 0 today 42 were 10–15 ng/mL, and from day 43 to day 365 levels were <10 ng/mL.

The experimental treatment protocol was tacrolimus and daclizumab (TAC/DAC). Two doses of daclizumab 2.0 mg/kg were administered; the first dose was given during the anhepatic period and the second dose between days 7 and 10. Biopsy confirmed that acute rejection was treated by increasing tacrolimus dose to attain trough levels of 15 ng/mL. If rejection persisted, thymoglobulin (RATG) was administered at 1.5 mg/kg for up to 3 days; acetaminophen and intravenous H1-receptor antagonists could be administered for premedication against anaphylactic reaction.

The reference treatment protocol was tacrolimus and steroids (TAC/STR). Steroids were given at a bolus dose of ≤500 mg in the perioperative period followed by tapered doses of 15–20 mg/day during month 1, 10–15 mg/day during month 2, 5–10 mg/day during month 3, then discontinued. Biopsy confirmed that acute rejection was treated by increasing tacrolimus dose to attain trough levels of 15 ng/mL. If there was no response, pulses of corticosteroids up to 1000 mg/day for 3 consecutive days could be administered.

Prophylactic antiviral treatment was required for cytomegalovirus (CMV) in cases where a CMV-positive donor graft was transplanted in a CMV-negative recipient.

Treatment with the following drugs was prohibited during the study: any other immunosuppressive agents except for mycophenolate mofetil (MMF) used at the discretion of the investigator for rejection treatment and polyclonal antibodies and OKT3 that were allowed for the treatment of intractable rejection.

### 2.3. Outcomes

Viral load of HCV at 12 months was the study primary endpoint. Quantitative and as necessary qualitative serum HCV-RNA evaluations were performed using branched DNA technology. In cases where a viremia reading was <3200 copies/mL, the TMA method was used as a sensitive analysis to measure copies ≥50/mL. Serum HCV RNA has been demonstrated to be a reliable indicator of hepatitis C liver disease [18].

The incidence of and time to hepatitis C recurrence was evaluated. HCV recurrence was diagnosed by liver biopsy and was performed as clinically indicated (in patients with elevated liver enzymes) or at protocol-defined biopsy control. Recurrence was defined as acute hepatitis with lobular necroinflammatory activity and/or as chronic hepatitis with lymphoplasmacytic portal inflammation with interphase hepatitis, lobular necroinflammatory activity, and with portal fibrosis. The histological grading and staging of biopsies for HCV evaluation were based on the modified HAI score by Ishak et al. [19]. Other outcomes measured were: the incidence of and time to first biopsy-proven acute rejection (BPAR); patient and graft survival at 12 months; renal function that was assessed by calculated creatinine clearance (Cockcroft-Gault formula [20]); the incidence of adverse events that were classified using MedDRA before database closure. Patients were followed up to 12 months even if prematurely discontinuing study treatments.

Samples for the evaluation of qualitative and quantitative serum HCV-RNA were collected at baseline and at subsequent regular assessments. Routine hematology and blood chemistry were assessed at 9 regular assessment visits (baseline (day 0, defined as the day of liver transplantation), days 1, 7, and 14, and months 2, 3, 6, 9, and 12) or at the time of premature study discontinuation. Tacrolimus trough levels were measured after the first dose then 2-3 times per week during the period of hospitalization and at each scheduled follow-up out-patient visit or more frequently if clinically indicated. Protocol biopsies were performed at the end of months 6 and 12; central assessment of biopsy specimens was conducted in a blinded manner. The evaluation of non-protocol-defined biopsies was done locally.

### 2.4. Sample Size and Randomization

There were no historical data on HCV viral load available at the time of the design of this exploratory study for the planned patient population. Therefore, sample size was based on a compromise between the considerations of feasibility and reasonable statistical power to detect a signal for a positive effect of the avoidance of steroids on HCV viral load.

Based on a (1-sided) Mann-Whitney *U* test and assuming a standard deviation of 0.6 log_10_ (copies/mL) the sample size of 50 evaluable patients per treatment arm was considered large enough to achieve a power >80%. Assuming a rate of 15% nonevaluable patients, 120 patients (60 per treatment arm) were to be randomized.

Allocation to treatment arms was performed using sealed sequentially numbered randomization envelopes provided by the study sponsor. RANCODE (version 3.6) was used to generate the randomization sequence. Randomization to treatment was 1 : 1 with stratification by center.

### 2.5. Statistical Methods

HCV viral load at 12 months was analyzed after a transformation using log_10_, which resulted in the unit log_10_ (IU/mL). The 1-sided Wilcoxon rank sum test was used to test for the superiority of the steroid-free arm (TAC/DAC) over the reference arm (TAC/STR). Kaplan-Meier analysis was used to estimate rates of patient and graft survival and survival over time to recurrence of HCV.

The significance level was chosen as *α* = 10% (1-sided). The chi-square test was used to compare differences in adverse events between treatment arms. The last-value-carried-forward method was used for replacement of missing laboratory values.

At investigator review of the results it became apparent that some patients had received antiviral treatment for HCV during the study. It was decided to perform post hoc analyses of HCV viral load at month 12 excluding patients who had taken antiviral medications for HCV. It was assumed that the elimination of these patients from an analysis of the primary endpoint would provide more information on the effect of the two protocols on HCV recurrence.

The full analysis set (FAS) population included all randomized patients who received at least one dose of any immunosuppressive medication according to the assigned study arm. The primary analysis set (PAS) comprised patients included in the FAS and with an HCV viral load above the limit of quantification at baseline (≥615 IU/m/L) to provide a population for analysis with active hepatitis C. Primary endpoint results are presented for the PAS and FAS populations, and the FAS is used for the presentation of all other results.

## 3. Results

### 3.1. Recipient, Donor, and Transplant Characteristics

**Figure 1:**
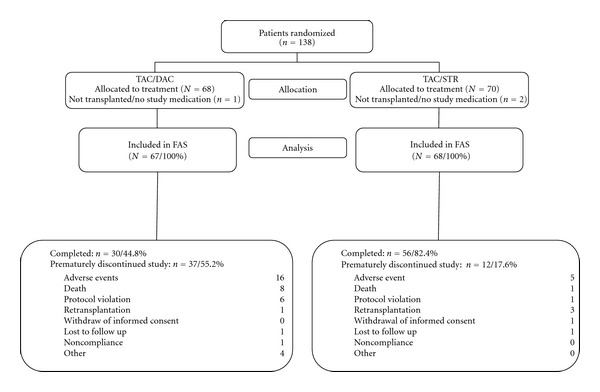
Progress of liver transplant recipients through the phases of the randomized study comparing a tacrolimus-based protocol with and without steroids. The rate of study completion was lower with TAC/DAC than with TAC/STR. Most commonly, patients in the TAC/DAC arm prematurely discontinued the study due to an adverse event. TAC: tacrolimus; DAC: daclizumab; STR: steroids; FAS: full analysis set; PAS: primary analysis set.

The flow of patient progress through the study is outlined in Figure 1. Of 138 patients randomized to receive treatment, 135 were included in the FAS population. The rate of study completion was lower in the TAC/DAC arm at 45% (30 of 67 patients) than the rate of 82% (56 of 68 patients) in the TAC/STR arm. The main reason for premature withdrawal in both arms was an adverse event (Figure 1). Study discontinuation during week 1 was more common in the TAC/DAC arm (12 of 37 withdrawn patients) than in the TAC/STR arm (4 of 12 withdrawn patients). An erroneously administered steroid bolus at transplantation was the reason for 5 of the 6 protocol violations in the TAC/DAC group and a patient in the TAC/STR arm violated the protocol for receiving basiliximab. A baseline HCV viral load below the limit of quantification was documented in 17 patients in the TAC/DAC arm and in 16 patients in the TAC/STR arm and led to exclusion of these patients from the PAS. The PAS, therefore, comprised 50 patients in the TAC/DAC and 52 patients in the TAC/STR arm. Of these, 19 patients (38%) in the TAC/DAC arm and 35 patients (67%) in the TAC/STR arm completed the study and were used in the analysis of the primary endpoint.

Baseline patient demographics were broadly similar between arms including the model for end-stage liver disease (MELD) mean scores (Table 1). All patients presented with a primary diagnosis of cirrhosis following hepatitis C infection. Hepatocellular carcinoma was present in 29 of 67 patients (43%) in the TAC/DAC arm and 34 of 68 patients (50%) in the TAC/STR arm at randomization. As would be expected in this European sample, the most common HCV genotype in both arms was 1b. All allografts were recovered from deceased donors. A greater number of organs were from male donors in the TAC/DAC arm than in the TAC/STR: this difference was not considered to affect study results.

Patients in both arms received antiviral treatment during the study in contradiction to the study protocol. This was 10 patients in the TAC/DAC arm and 21 in the TAC/STR arm included in the FAS and 8 patients in the TAC/DAC arm and 20 in the TAC/STR arm included in the PAS. Antiviral treatment was administered to patients with biopsy-proven recurrence to reduce the risk of early severe HCV recurrence, which is associated with increased risk for graft loss.

### 3.2. Immunosuppression

Mean tacrolimus trough levels in both arms were well within the targeted range of 10–15 ng/mL up to day 42. Mean trough levels at month 12 were 7.6 ng/mL in the TAC/DAC arm and 8.6 ng/mL in the TAC/STR arm, which were within the targeted range of <10 ng/mL. The mean (SD) daily dose of tacrolimus at month 12 was 0.1 (0.06) mg/kg in the TAC/DAC arm and 0.05 (0.04) mg/kg in the TAC/STR arm.

All patients in the TAC/DAC arm received one dose of daclizumab and 52 of 67 (78%) received a second dose.

In violation of the protocol, 6 of 67 patients (9%) in the TAC/DAC arm erroneously received steroid boluses perioperatively; the median (range) dose was 7.7 (1.9–9.8) mg/kg. A few patients (1–3 patients at each scheduled assessment visit) were taking maintenance steroids. In the TAC/STR arm, 56 of 68 patients were maintained on steroids at 3 months, which decreased to 45 patients at 6 months and to 17 patients at 12 months. The median daily dose of maintenance steroids at 12 months was 0.04 (0.01–0.15) mg/kg. Pulse steroid treatment for acute rejection was administered to 3 patients in the TAC/DAC arm and to 10 patients in the TAC/STR arm.

### 3.3. HCV Viral Load

There was no significant difference between the treatment arms in HCV viral load at month 12 (Table 2). Results of the analysis using the PAS and the FAS were similar.

A post hoc analysis of HCV viral load that was performed on patients who had not received antiviral medications during the study showed comparable median values for HCV viral load for the PAS (Table 2). The difference in median values between the treatment arms reached statistical significance for the FAS. In the interpretation of these results it is important to note that analyses were performed post hoc on patients completing the study and the number of study completers was lower in the TAC/DAC arm.

### 3.4. HCV Recurrence

**Figure 2:**
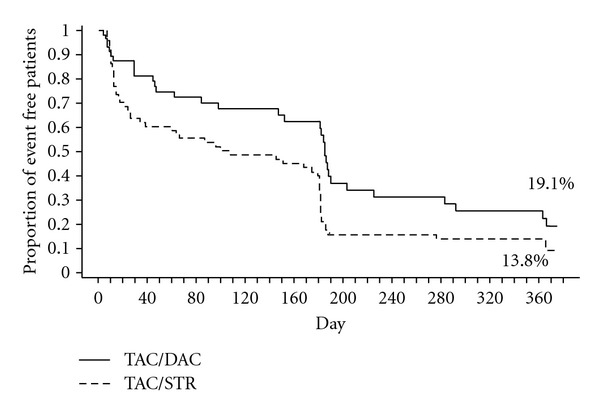
Estimated rate of patients free from recurrence of HCV Infection (Kaplan-Meier Method) at 12 months as confirmed by central biopsy. Freedom from HCV recurrence at 12 months was 19.1% with the TAC/DAC steroid-free protocol and 13.8% with the TAC/STR protocol (Kaplan-Meier method) with a significant difference in survival curves between treatments (95% CI, −0.105 to 0.211%; *P* = 0.020, Wilcoxon Gehan test). Protocol biopsies were performed at months 6 and 12 accounting for the higher number of events reported at these time points. TAC: tacrolimus; DAC: daclizumab; STR: steroids.

The rate of patients free of HCV recurrence at 12 months was 19.1% with the TAC/DAC steroid-free protocol and 13.8% with the TAC/STR protocol (Kaplan-Meier method) with a significant difference in survival curves between treatments (95% CI, −0.105 to 0.211%; *P* = 0.020) (Figure 2). Early HCV recurrence at month 3 was less common with the steroid-free protocol at 55% compared with 70% in the steroid arm.

Results of the post hoc analysis, in which liver biopsies were centrally reviewed and HCV recurrence was censored for antiviral treatment, favored the TAC/DAC immunosuppression protocol. The estimated rate of patients free of HCV recurrence in this analysis was significantly higher in the TAC/DAC arm at 20.2% compared with 13.1% in TAC/STR (95% CI, −0.091 to 0.234%; *P* = 0.022) (Table 3).

### 3.5. Fibrosis Stage

Protocol biopsies (performed at months 6 and 12) and nonprotocol biopsies were centrally reviewed to assess fibrosis score. As depicted in Table 3, there were no differences between the treatment arms in the total mean modified fibrosis staging score; mean scores were <2 in both arms. More cases of severe fibrosis, as evidenced by a score of ≥4, were assessed in the TAC/DAC arm (4 of 35 biopsies) compared with the TAC/STR arm (0 of 54 biopsies). There was no clinically relevant difference between the treatment arms in mean total modified HAI grading score at 12 months (Table 3).

### 3.6. Patient and Graft Survival

The overall estimated rate of patient survival (Kaplan-Meier method) was significantly lower in the TAC/DAC arm (95% CI, −0.227 to −0.019%; *P* = 0.025) (Table 3).

The estimated rate of graft survival was numerically lower in the TAC/DAC arm (Table 3). The rate of graft loss was 19.4% in the TAC/DAC arm (13 of 67 patients) and 8.8% in the TAC/STR arm (6 of 68 patients). Patient death accounted for 11 of the 13 grafts lost in the TAC/DAC arm and for 3 of the 6 grafts lost in the TAC/STR arm.

There were 11 patient deaths in the TAC/DAC arm; 8 deaths occurred during the study and 3 after premature withdrawal. The causes of death were HCV recurrence (3 patients), cardiovascular complications (5 patients), surgical complications (2 patients), and infection (1 patient). Specifically, cardiac complications were myocardial ischemia, cardiac tamponade, cerebral hemorrhage, cardiac failure (identified as not or unlikely related to study drug), and cerebrovascular accident (identified as possibly related to study drug). There were 3 patient deaths in the TAC/STR arm, 1 death during the study and 2 after study withdrawal. The causes of death were HCV recurrence, surgical complications, and infection (each 1 patient).

### 3.7. Biopsy-Proven Acute Rejection

The overall frequency of BPAR was significantly lower in the TAC/DAC than in the TAC/STR arm (*P* = 0.048, chi-square test) (Table 3). Estimated freedom from BPAR (Kaplan-Meier method) was numerically but not significantly higher with TAC/DAC (78.4%) than with TAC/STR (66.1%) (95% CI, −0.042 to 0.287%) (Table 3).

Histological findings showed that the majority of rejections were classified as mild (Banff I) in both treatment arms: 8 of 11 (11.9%) in the TAC/DAC arm and 14 of 21 (20.6%) in the TAC/STR arm. One rejection in the TAC/DAC arm was classified as severe (Banff III).

### 3.8. Renal Function

Baseline serum creatinine was comparable. Renal function at 12 months, assessed using calculated creatinine clearance (Cockroft-Gault method) and serum creatinine, was comparable between the two arms. Mean (SD) creatinine clearance was 72.2 (27.3) mL/min in the TAC/DAC arm and 74.7 (30.6) mL/min in the TAC/STR arm. Mean serum creatinine in the TAC/DAC arm was 128.4 (68.8) *μ*mol/L and 110.8 (59.7) *μ*mol/L in the TAC/STR arm.

### 3.9. Adverse Events

A summary of the adverse event profile for the two treatment arms is presented in Table 4. An adverse event occurred in 91% of TAC/DAC and 97% of TAC/STR patients. Serious adverse events occurred in two-thirds of patients, most commonly infection. There was a significantly lower incidence of hepatitis C reported as an adverse event in the TAC/DAC arm than in the TAC/STR arm and a significantly higher incidence of thrombocytopenia in the TAC/DAC arm than in the TAC/STR arm.

The number of patients discontinuing the study due to a treatment emergent adverse event was threefold higher in the TAC/DAC arm than in the TAC/STR arm. In the TAC/DAC arm, 16 of 67 patients (24%) discontinued the study compared with 5 of 68 patients (7.4%) in the TAC/STR arm.

Malignancies occurred with an incidence of 4.5% (3 of 67 patients) in the TAC/DAC arm and 3% (2 of 68 patients) in the TAC/STR arm. These were 2 cases of hepatocellular carcinoma and 1 case of uterine cancer in the TAC/DAC arm and 1 basal cell carcinoma and 1 peritoneal carcinoma in the TAC/STR arm. The incidence of new onset diabetes mellitus after transplantation was 1.5% (1 patient) in the TAC/DAC arm and 6% (4 patients) in the TAC/STR arm.

## 4. Discussion

The results of this study showed that the steroid-free protocol in comparison to the protocol with steroids had a negligible impact on viral HCV RNA values at 12 months. Results of the post hoc analysis, which excluded patients who had taken antiviral medications for treatment of HCV, indicated an even smaller between-treatment difference in viral load in the primary analysis set but a significant difference in the full analysis set.

This study, despite its limitations, is one of a few randomized controlled multicenter studies to prospectively analyze HCV viral load and disease recurrence using a steroid-free protocol. While we did not observe an impact of this experimental treatment protocol on viral load, we did find that the estimated rate of patients remaining free of HCV recurrence was slightly better with the steroid-free protocol. The exclusion of patients who had received antiviral treatment during the study did not affect rates of HCV recurrence in either arm or the between-treatment arm difference in rates. As reported in other comparative trials in HCV-positive patients [10, 15, 21], we observed a tendency for earlier recurrence in the steroid arm. The higher number of patients in that arm who received steroid pulse treatments for acute rejection might have contributed to this result. Conversely, we observed a tendency for more advanced fibrosis occurring with TAC/DAC. Wiesner et al. [1] recommend yearly biopsies beyond 5 years followingtransplantation to determine histological progression of HCV infection. A longer-time span to register fibrotic changes and a more detailed analysis of fibrosis progression might have yielded more clinically relevant comparative data of longer term outcomes with the two treatment protocols.

Using daclizumab in place of steroids had no compromising effect on efficacy as shown by the lower incidence of BPAR with TAC/DAC than TAC/STR. Adding MMF to the steroid-free protocol may have provided higher immunocoverage with lower BPAR incidence as reported in a similar study using a triple-drug steroid-free protocol [21]. We acknowledge that many features of mild rejection and HCV recurrence are shared and that mild rejection in the presence of HCV might be difficult to distinguish from HCV recurrence alone.

There were unexpected differences between the treatment arms in patient tolerability of treatment and the incidence of premature withdrawal. Although adverse events occurred with a similar frequency in the two treatment arms, an adverse event was more commonly the cause of premature study withdrawal in the steroid-free arm. No single type of adverse event accounted for the higher number of dropouts. In a parallel arm randomized trial with treatment interventions similar to those we applied, premature study discontinuations were also higher in the tacrolimus + daclizumab than in the tacrolimus + steroids arm [17]. The higher incidence of thrombocytopenia in the TAC/DAC arm is difficult to explain as other consequences of bone marrow suppression (pancytopenia and leukopenia) were higher in the TAC/STR arm and incidences of anemia were similar between treatment arms. Lower rates of hepatitis C reported as an adverse event in the TAC/DAC arm may indicate a potential safety benefit of this protocol over one with steroids.

Patient survival was lower, albeit not significantly, in the steroid-free than in the steroid arm but is comparable to results reported from another steroid-free clinical trial [15]. We cannot provide an explanation as to why this occurred. We could find no demographic or comorbidity factors, or common events leading to or causing death or serious adverse events to account for this difference. The higher number of patient deaths caused by cardiac events in the steroid-free arm could be attributed to chance. Contrastingly, patient survival in the steroid arm was 96%, which is exceptionally good considering that the study population was HCV positive. This survival rate is higher than the rate of 88.4% for unadjusted 1-year patient survival (deceased donor) reported in the 2009 OPTN/SRTR Annual Report on the status of liver transplantation [22].

We urge caution in the interpretation of these results for several reasons. The first is the low number of patients providing data for the primary endpoint; this affected the statistical power of the study generating inconclusive results. Secondly, only the post hoc analysis, which is biased due to the elimination of patients treated with antiviral agents and limited because of the post hoc method, revealed superiority of the steroid-free protocol over the reference protocol in impacting on HCV viral load in HCV-positive patients.

It is difficult to recommend a steroid-free protocol for HCV-positive patients based on study results. The impact of this protocol on HCV viral load at one year was insignificant, and although there was a tendency for later HCV recurrence and lower incidences of rejection, we also observed a higher dropout rate and a lower patient survival rate with tacrolimus and daclizumab compared to tacrolimus and steroids. A study of longer duration could provide important clinical information on the relationship between type of treatment protocol and viral load, HCV recurrence, and fibrosis progression.

## Figures and Tables

**Table 1:** Baseline recipient and donor characteristics of the FAS population.

	TAC/DAC	TAC/STR
	*N* = 67	*N* = 68
Recipient age, mean (SD)	53.1 (9.6)	55.3 (6.5)
Male, no. (%)	49 (73.1)	45 (66.2)
Mean (SD) MELD score	16.2 (6.3)	14.5 (6.1)
HCV genotype:		
1	7 (12.1)	5 (8.3)
1a	8 (13.8)	4 (6.7)
1b	28 (48.3)	37 (61.7)
2	1 (1.7)	1 (1.7)
2a/c	1 (1.7)	0
3	4 (6.9)	5 (8.3)
3a	6 (10.3)	7 (11.7)
4	2 (3.4)	1 (1.7)
4a	1 (1.7)	0
HCV-RNA positive*, no. (%)	50 (84.7)	55 (94.8)
Serum HCV-RNA, median (min; max) IU/mL	5.05 (0.95; 6.61)	5.09 (0.95; 6.12)
Donor age, mean (SD) years	49.1 (19.0)	50.6 (19.3)
Donor age <60 years, mean (SD) years	41 (61.2)	47 (64.7)
Donor age ≥60 years, mean (SD) years	26 (38.8)	24 (35.3)
Donor sex, male, no. (%)	43 (64.2)	32 (47.1)
Traumatic cause of donor death, no. (%)	21 (33.3)	20 (30.8)
Non-traumatic cause of donor death, no. (%)	42 (66.7)	45 (69.2)
Ischemic time, mean (SD), hours	8.8 (3.3)	8.0 (3.0)
Donor/recipient CMV serological status:		
Donor +/recipient −, no. (%)	8 (11.9)	6 (8.8)
ABO identical, no. (%)	66 (98.5)	64 (94.1)

SD: standard deviation; HCV: hepatitis C virus; MELD: mean model for end-stage liver disease; CMV: cytomegalovirus.

*HCV-RNA above the limit of quantification at baseline.

**Table 2:** Measurement of median (min; max) HCV viral load at month 12 in patients completing the study*.

	*N*	TAC/DAC	*N*	TAC/STR
Median (min; max) HCV viral load, IU/mL; PAS	19	5.46 (0.95; 6.54)	35	5.91 (0.95; 6.89)
Median (min; max) HCV viral load, IU/mL; PAS (no viral) treatment during study	14	5.77 (3.45; 6.54)	20	5.99 (5.32; 6.89)
Median (min; max) HCV viral load, IU/mL; FAS	25	4.52 (0.95; 6.4)	46	5.9 (0.95; 6.89)
Median (min; max) HCV viral load, IU/mL; FAS (no viral) treatment during study	20	5.25 (0.95; 6.54)^†^	26	5.99 (3.47; 6.89)

PAS: Primary analysis set: All randomized and transplanted subjects with baseline viral load above the limit of quantification.

FAS: Full analysis set: All randomized and transplanted patients

*Patients completing the study with data available within ± 14 days of day 365.

^†^ *P* = 0.024 Wilcoxon rank sum test for superiority of TAC/DAC over TAC/STR.

**Table 3:** Secondary study endpoints-full analysis set.

	TAC/DAC		TAC/STR	
	*N*		*N*	
Estimated rate of patients free of HCV recurrence*, %	67	19.1	68	13.8
Estimated rate of patients free of HCV recurrence, censored for antiviral treatment, %	67	20.2	68	13.1
Modified fibrosis staging^†^, mean score (SD)	35	1.9 (1.2)	54	1.6 (0.7)
Stage 0, no. (%)		0		1 (1.7)
Stage 1, no. (%)		18 (41.9)		25 (43.1)
Stage 2, no. (%)		10 (23.3)		22 (37.9)
Stage 3, no. (%)		3 (7.0)		6 (10.3)
Stage 4, no. (%)		2 (4.7)		0
Stage 5, no. (%)		2 (4.7)		0
Stage 6, no. (%)		0		0
Modified HAI grading^‡^, mean total score (SD)	35	6.7 (2.7)	54	6.5 (2.5)
Patient survival^§^, %	67	83.1	68	95.5
Graft survival, %	67	80.1	68	91.1
Biopsy-proven acute rejection^¶^, no. (%)	67	11 (16.4)	68	21 (30.9)
Treatment resistant, no. (%)		4 (6.0)		2 (2.9)
Treatment sensitive, no. (%)		3 (4.5)		7 (10.3)
Spontaneous resolution		4 (6.0)		14 (20.6)
Estimated freedom from biopsy-proven acute rejection^¶^, %	67	78.4	68	66.1

HCV: hepatitis C virus; SD: standard deviation; HAI: histologic activity index.

*95% CI, −0.105 to 0.211%; *P* = 0.020, Wilcoxon Gehan test.

^†^Worst fibrosis staging per patient using the modified scoring system of Ishak et al. [19] in which a score of 6 is complete fibrosis. Central evaluation of nonprotocol biopsies and protocol biopsies (at 6 and 12 months) was used to assess fibrosis score.

^‡^Worst histological HCV grading per patient using the modified HAI grading [14]. Central evaluation of nonprotocol biopsies and protocol biopsies (at 6 and 12 months) was used to assess HAI grading.

^§^95% CI (Greenwood formula) for the difference in 12-month patient survival was −0.227 to −0.019%, *P* = 0.025.

^¶^Local evaluation of biopsies. *P* = 0.048, chi-square test comparing the numbers of patients.

**Table 4:** Incidence of adverse events occurring in ≥10% in either arm of full analysis set and with a significant difference between treatment arms, no. (%).

	TAC/DAC	TAC/STR
	*N* = 67	*N* = 68
Hepatitis C*	28 (41.8)	43 (63.2)
Hyperglycemia	9 (13.4)	13 (19.1)
Hyperkalemia	8 (11.9)	7 (10.3)
Anemia	22 (32.8)	21 (30.9)
Thrombocytopenia^†^	19 (28.4)	6 (8.8)
Leukopenia	10 (14.9)	15 (22.1)
Pancytopenia	4 (6.0)	7 (10.3)
Renal failure	20 (29.9)	23 (33.8)
Acute renal failure	7 (10.4)	6 (8.8)
Diarrhea	9 (13.4)	14 (20.6)
Ascites	3 (4.5)	8 (11.8)
Hypertension	17 (25.4)	20 (29.4)
Hypotension	8 (11.9)	9 (13.2)
Pleural effusion	12 (17.9)	15 (22.1)
Headache	8 (11.9)	8 (11.8)
Tremor	9 (13.4)	6 (8.8)
Peripheral edema	7 (10.4)	10 (14.7)
Insomnia	7 (10.4)	5 (7.4)
Back pain	8 (11.9)	9 (13.2)
Pruritus	5 (7.5)	7 (10.3)

Not presented are adverse events related to the transplanted allograft or procedural complications.

*P = 0.016; ^†^ *P* = 0.004 (Fisher's exact test comparing the number of patients).
